# Parahippocampal deactivation and hyperactivation of central executive, saliency and social cognition networks in autism spectrum disorder

**DOI:** 10.1186/s11689-022-09417-1

**Published:** 2022-01-25

**Authors:** Susana Mouga, Isabel Catarina Duarte, Cátia Café, Daniela Sousa, Frederico Duque, Guiomar Oliveira, Miguel Castelo-Branco

**Affiliations:** 1 CIBIT - Coimbra Institute for Biomedical Imaging and Translational Research, University of Coimbra, Azinhaga de Santa Comba, 3000-548 Coimbra, Portugal; 2 ICNAS - Institute of Nuclear Sciences Applied to Health, University of Coimbra, Coimbra, Portugal; 3 Neurodevelopmental and Autism Unit from Child Developmental Center, Hospital Pediátrico, Centro Hospitalar e Universitário de Coimbra, Coimbra, Portugal; 4 Faculty of Medicine, University of Coimbra, Coimbra, Portugal

**Keywords:** Autism spectrum disorder, Central executive network, Saliency network, Social cognition network, fMRI, Ecological task

## Abstract

**Background:**

The concomitant role of the Central Executive, the Saliency and the Social Cognition networks in autism spectrum disorder (ASD) in demanding ecological tasks remains unanswered. We addressed this question using a novel task-based fMRI virtual-reality task mimicking a challenging daily-life chore that may present some difficulties to individuals with ASD: the EcoSupermarketX.

**Methods:**

Participants included 29 adolescents: 15 with ASD and 15 with typical neurodevelopment (TD). They performed the EcoSupermarketX (a shopping simulation with three goal-oriented sub-tasks including “no cue”, “non-social” or “social” cues), during neuroimaging and eye-tracking.

**Results:**

ASD differed from TD only in total time and distance to complete the “social cue” sub-task with matched eye-tracking measures. Neuroimaging revealed simultaneous hyperactivation across social, executive, and saliency circuits in ASD. In contrast, ASD showed reduced activation in the parahippocampal gyrus, involved in scene recognition.

**Conclusions:**

When performing a virtual shopping task matching the performance of controls, ASD adolescents hyperactivate three core networks: executive, saliency and social cognition. Parahippocampal hypoactivation is consistent with effortless eidetic scene processing, in line with the notion of peaks and valleys of neural recruitment in individuals with ASD. These hyperactivation/hypoactivation patterns in daily life tasks provide a circuit-level signature of neural diversity in ASD, a possible intervention target.

## Background

Autism spectrum disorder (ASD) is a heterogeneous neurodevelopmental disorder characterized by a varied severity of deficits in social communication and interaction, and repetitive patterns of behaviour and interests [1].

Considering ASD symptoms, two brain circuits have received particular isolated attention: the social brain and central executive networks (CEN), while the salience network may also deserve further investigation in this context.

The circuit that supports social cognition includes the prefrontal cortex (PFC), amygdala, anterior thalamus, anterior cingulate cortex (ACC), posterior cingulate cortex (PCC), superior temporal sulcus (STS) and temporo-parietal junction (TPJ) [2–6]. Using tasks operationalizing social cognition, some studies reported distinct activation of these regions in individuals with ASD [7–10], combined with structural differences in areas such as STS, insula, fusiform face area and inferior frontal gyrus [10]. There is growing consensus that the impairments in ASD require multiple-circuit levels of analysis [4, 11–13].

Although functional investigations of ASD pathophysiology have focused on social cognition, impairments in other behavioural and cognitive domains are present, such as alterations in the relative perceptual salience of social and non-social stimuli. For example, differences in executive functioning have also been reported in individuals with ASD [14–17]. How changes in these cognitive domains relate to social cognition and other impairments remains an open question. These alterations, in particular the impairments in executive functioning (EF), have an early onset, can aggravate with age and persist despite amelioration of other ASD symptoms [1, 18].

EF encompasses cognitive skills, including planning, working memory, attention, inhibition, self-monitoring, self-regulation and initiation [19]. These high-level cognitive processes entail the modulation of lower-level processes, enabling flexible adaptive behaviour [20]. In individuals with ASD, EF is impaired early on and is thought to have a significant influence on social cognition abilities and adaptive behaviour and to be major contributor to everyday deficits, disability and absence of social autonomy [15, 21–23].

Functional neuroimaging of EF in ASD suggested the involvement of the dorsolateral prefrontal cortex (DLPFC) [24–26], superior and inferior parietal lobules [26–30] and anterior frontal areas [29]. Distinct results may depend on the task used, contextual demands, group heterogeneity and ASD comorbidities [20, 31]. A recent meta-analysis revealed that both (typical neurodevelopment)TD and participants with ASD recruited PFC regions during EF. ASD presented greater activation in ACC and lesser activation in the inferior parietal lobule (IPL), left middle frontal gyrus and left medial prefrontal cortex (MPFC) [32].

ASD has also been linked to alterations in the salience network [33], although evidence is more limited. This circuit is involved in selecting the attentional focus, playing a role in switching between internally (for example, the default mode network) and externally focused processing nodes (for example the central executive network) [34–37].

Most of the neuroimaging studies of the salience network in individuals with ASD focused on the resting-state functional connectivity and showed inconsistent results [11, 38–40]. It has been proposed that ASD and TD participants can be discriminated based on hyperconnectivity within the salience network [41]. However, little is known about how this altered resting-state connectivity relates to brain activity during information processing [42]. Task-based experimental designs are therefore needed.

Furthermore, it is still poorly understood how the executive network interrelates with the salience network in health and disease and how it functionally connects with social networks. For example, TPJ, which is related to the theory of mind and the distinction between self and other, is also associated with the direction of attention for salient cues.

Cognitively demanding goal-directed tasks in the human brain are thought to involve the dynamic interplay of these large-scale neural networks, in particular the salience network and the CEN [43]. This raises the question how individuals with ASD recruit these networks when attempting to successfully perform real-life tasks.

In this study, we investigated task-driven group differences with a focus on EF and its connected networks. We expected higher activity in EF networks, posing a challenge to the other networks and in particular, the saliency hubs because of their switching role between task-positive and other networks. To achieve this goal, we used an fMRI design to study brain activity of ASD and TD participants while performing a task that required strong executive function demands. Cue saliency processing in the context of a virtual ecological social situation was also a feature of this goal-oriented task. We hypothesized that this approach would show the group differences in central executive network and its concomitant impact on related salience and social brain networks.

We recorded fMRI data while participants performed a task developed at our Lab: the EcoSupermarketX [44], a virtual reality task, monitored with eye-tracking, consisting of going shopping to a supermarket, with three types of sub-tasks that include social cues, non-social cues, or no cues. EcoSupermarketX was based in two main premises: on the one hand, shopping is a good example of a real-world task that often draws heavily on EF, contextual cueing and social/non-social cognition, and on the other hand, different assessment and rehabilitation studies of ASD populations have successfully used supermarket settings [45, 46].

## Methods

### Participants

A total of 34 participants took part in the study: 18 individuals with ASD without intellectual disability and 16 TD controls. Due to exclusion criteria concerning excessive head movement during the fMRI acquisition, three participants with ASD and two with TD were excluded from the analysis. As a result, 15 individuals with ASD (14 male and 1 female; median age = 16 years 4 months) and 14 chronological age-matched control participants (12 male and 2 female; median age = 15 years 2 months) were included for the final analysis.

The participants with ASD were recruited from the Unit of Neurodevelopment and Autism, from the Child Developmental Centre of the Paediatric Hospital of Coimbra. The selection was based on chronological age (≥ 10 years) and on the ability to cooperate in the fMRI acquisition.

ASD diagnosis was assigned on the basis of the gold standard instruments: parental or caregiver interview—Autism Diagnostic Interview—Revised, ADI-R [44, 47], direct structured proband assessment—Autism Diagnostic Observation Schedule, ADOS [48, 49], and clinical examination performed by an experienced neurodevelopmental Paediatrician, based on the current diagnostic criteria for ASD from the Diagnostic and Statistical Manual of Mental Disorders 5, DSM-5 [1]. All participants with ASD had positive results in the ADI-R and ADOS for autism or ASD and met the criteria for ASD from the DSM-5. A comprehensive medical observation excluded associated medical conditions such as epilepsy, neurocutaneous or other genetic syndromes or other usual comorbidities in ASD samples, such as Attention Deficit Hyperactivity Disorder or intellectual disability.

The TD group included 14 participants with typical neurodevelopment who were matched for chronological age, performance intelligence quotient [50], sex and handedness with the ASD group (Mann-Whitney *U* or Pearson’s chi-square test, *p* > .05). The Social Communication Questionnaire which is a screening test for ASD symptoms was completed by the TD group participants’ parents to exclude ASD [51]. The positive cut-off for ASD is equal to or above 15 and all participants scored below.

One participant with ASD and one TD participant demonstrated left-hand dominance as it was measured using the Edinburgh Inventory [52]. When necessary, correction to normal vision was ensured using specific eyeglasses compatible with the magnetic field. Given that some individuals with ASD exhibited hypersensitivity to the sound, we worked with each patient, so they were previously familiarized with the magnetic resonance imaging (MRI) sounds and were able to attend and perform experimental tasks inside the scanner. Nonetheless, all participants used hearing protection.

Both groups underwent a comprehensive neuropsychological evaluation and an assessment of the intelligence quotient (IQ) to exclude Intellectual disability (full-scale IQ > 70). All participants included in the study received the Portuguese adapted version of the Wechsler Intelligence Scale for Children—third edition (WISC-III) [53] or the Wechsler Adult Intelligence Scale—third edition (WAIS-III) [54], according to the participant’s age.

The demographic characterization of both groups is summarized in Table 1.

**Table 1:** Characterization of the ASD and TD groups

	ASD	TD	
	Median (IQR; min-max)	Median (IQR; min-max)	
**N**	15	14	
**Sex (M/F)**	14/1	12/2	*p* = .501*
**CA (years and months)**	16 y 5 m (4 y 5 m; 12 y 2 m–22 y 4m)	15 y 9 m (2 y 8 m; 10 y 8 m–18 y 6 m)	*p* = .290*
**Handedness (R/L)**	14/1	13/1	*p* = .960*
**FSIQ**	100.0 (24; 71–137)	116.5 (24; 92–152)	*p* = .004
**VIQ**	97.0 (17; 78–126)	120.5 (29; 98–145)	*p* < .001
**PIQ**	101.0 (35; 73–136)	107.0 (21; 85–146)	*p* = .172*
**ADI-R RSI**	16.0 (11; 7–26)	–	
**ADI-R L/C**	10.0 (4; 3–22)	–	
**ADI-R RB/I**	4.0 (2; 2–11)	–	
**ADOS COM**	5.0 (2; 3–7)	–	
**ADOS SI**	8 (3; 4–14)	–	
**ADOS Total**	12.0 (5; 8–19)	–	

*Note. ASD*, autism spectrum disorder group; *TD*, typical neurodevelopment group; *IQR*, interquartile range; *min*, minimum; *max*, maximum; *M*, male; *F*, female; *CA*, chronological age; *R*, right; *L*, left; *FSIQ*, full-scale intelligence quotient (IQ); *VIQ*, verbal IQ; *PIQ*, performance IQ; *ADI-R RSI*, ADI-R Reciprocal Social Interactions; *ADI-R L/C*, Autism Diagnostic Interview—Revised Language/Communication; *ADI-R RB/I*, ADI-R Repetitive Behaviours/Interests; *ADOS COM*, ADOS Communication; *ADOS SI*, ADOS Social Interaction. *Mann-Whitney *U* or Pearson’s chi-square *p* > .05

### Procedure

The acquisition session comprised one structural sequence and three functional sequences. The EcoSupermarketX paradigm was presented on an LCD monitor (48.5 × 87.8 cm, 1920 × 1080-pixel resolution, 60-Hz refresh rate) which the participants viewed through a mirror mounted above their eyes. The participants could actively navigate the scenario and select the response using a magnetic resonance-compatible joystick (Hybridmojo, San Mateo CA, USA). During the acquisition session, eye-tracking data (sample frequency of 1K) were recorded inside the scanner using Eyelink 1000 software (EyeLink 1000 Plus, SR Research, Mississauga, Ontario, Canada).

### EcoSupermarketX

EcoSupermarketX is a non-immersive virtual reality task that aims to accurately evaluate the social cognition and EF abilities of the participants using a realistic quotidian scenario—a computer-generated supermarket (for the original version of the task, please see our previous work [55]). EcoSupermarketX was generated with Vizard Virtual Reality toolkit 5.2 (WorldViz, Santa Barbara, USA).

The task in the MRI was preceded by a similar task outside the scanner [55], so the participants were already familiarized with the stimuli. The task has a joystick that allowed the participants to navigate in the scenario and to rotate (as if they were turning the head and looking right or left).

**Fig. 1:**
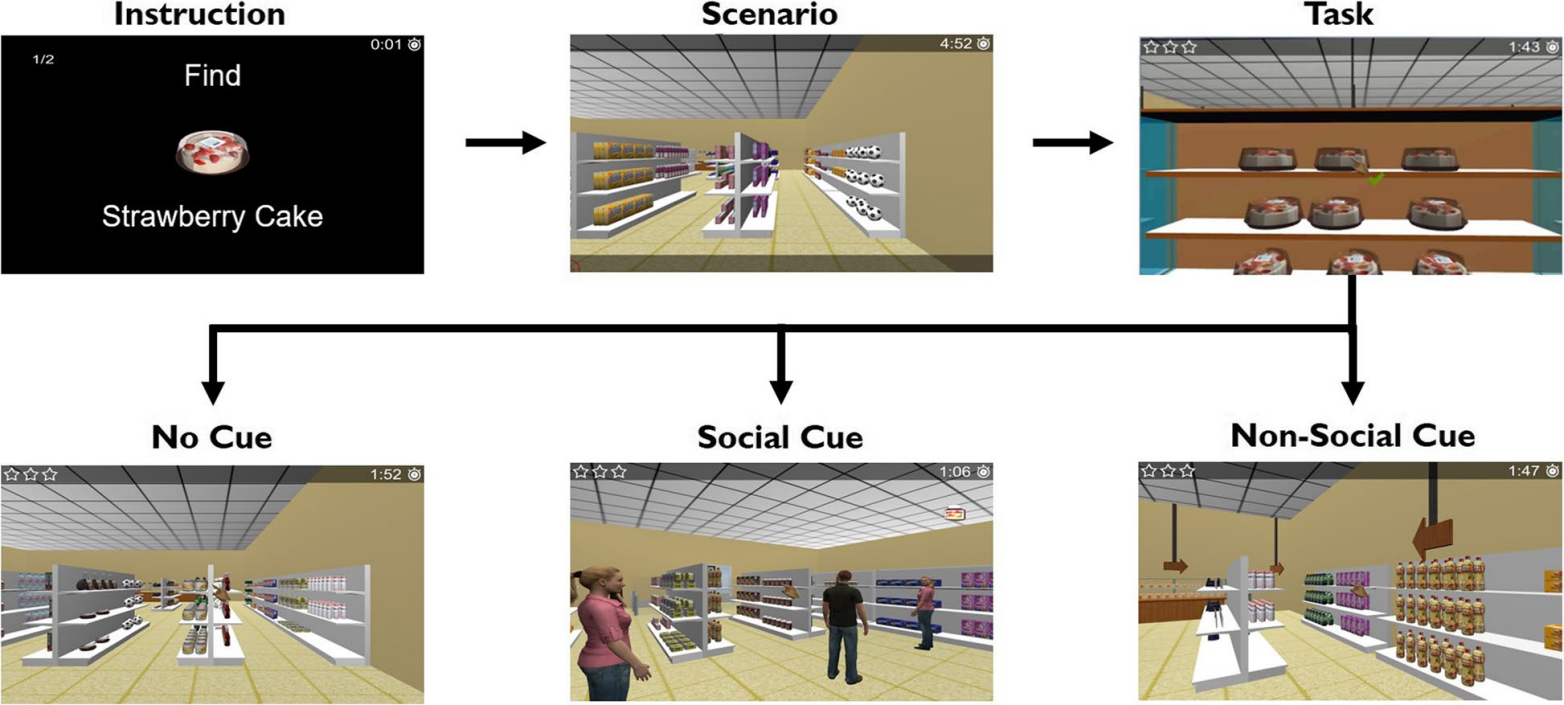
EcoSupermarketX task design, considering the different types of cues. The task blocks included an instruction that consisted in the grocery list the participants had to pick. The grocery list (3 items) was presented as an instruction individually in a trial-by-trial basis: “Find strawberry cake” followed by “Find sausages” and “Find Olive Oil”, for example (each item image and name appeared for 3 s). The groceries were replaced randomly in the shelves on a trial-by-trial basis. Participants were instructed to collect all items in the sequence they appeared in the list, and as fast and accurately as possible. Additionally, there were three different conditions related to cues: no cue, non-social cue (wooden arrow) and social cue (avatar gazing to the grocery). The task blocks were divided into three runs with an interval between each run

The experimental task consisted of three separate runs with variable duration (since it was dependent on the individual performance). At the end, a total of 18 trials (grocery list presentation + shopping) were performed. Each trial started with the grocery list (three items) presented as an instruction on a trial-by-trial basis: “Find strawberry cake” followed by “Find sausages” and then “Find olive oil”, for example (each item image and name appeared for 3 s, see Fig. 1). Then, the participant was instructed to search and pick those groceries from the supermarket shelves. The groceries were replaced randomly in the shelves on a trial-by-trial basis. For every single list with three items, which defines a trial, the participant had a maximum of 3 min to find the groceries and conclude the “shopping”. Nevertheless, the trial ends as the participant completes the list. Participants were instructed to collect all items in the sequence they appeared in the list, and as fast and accurately as possible. They had to plan and monitor their behaviour to complete the task successfully.

Additionally, participants were informed that during the task three situations could happen: they could have help from a person (an avatar)—social cue; an arrow—non-social cue; or no help at all—no cue. They were not told what specifically the person (avatar gazing to the grocery) was doing or what kind of arrows (wood arrow) were presented (Fig. 1). Participants performed six trials per cue type. There were 30 s of baseline (black screen with a central cross) and between the instruction (grocery list encoding) and the beginning of the shopping, there was is a variable interval of 12, 14 or 16 s.

To reduce merely memory constraints, an image of the requested item from the list was displayed in the upper right corner after 40 s of fruitless searching, giving the opportunity to the participant to conclude the trial. Focusing on the enhancement of realism and ecological validity, realistic three-dimensional (3D) forms and commercial brands were used to depict the groceries included in the supermarket scenario.

### Eye-tracking recording and measures

Eye movements were tracked using an infrared-emitting video-based eye tracker (EyeLink 1000 Plus, SR Research, Mississauga, Ontario, Canada). We used mono mode and pupil corneal reflection, at a 1K sample rate. The tracker has a reported gaze position accuracy of 0.25–0.50° and a spatial resolution of 0.05. A 9-point calibration procedure with a fixation circle was performed before each run. After the calibration, there was a validation trial to ensure the precision of the data collection (tracking error smaller than 1° visual angle). As participants were performing a dynamic virtual-reality task in which they were navigating around the supermarket, the frames in the screen were always different for all participants. In this way, the areas of interest (AOI) were defined in the virtual-reality software, which received the participants’ gaze coordinates from the eye-tracking software in a real-time mode. Using those screen coordinates, we computed the time that the participant was looking at each AOI on a real-time basis. The AOI related to the different types of cues were defined: head (because the avatar is looking at the item) and arrow.

### fMRI data acquisition and pre-processing

MRI experiments were performed on a 3 Tesla Magnetom Prisma Fit scanner (Siemens, Erlangen, Germany), using a 64-channel head coil. The scanning session included one T1-weighted 3D anatomical magnetization-prepared rapid acquisition gradient echo pulse sequence, with repetition time (TR) = 2530 ms, echo time (TE) = 3.5 ms, resolution 1 mm^3^ (voxel size 1.0 × 1.0 × 1.0 mm), flip angle = 7°, 192 slices, field of view (FOV) = 256 × 256 mm and a slice thickness of 1mm.

Afterwards, three functional runs were acquired using a T2*-weighted gradient echo-planar imaging sequence, with a slice thickness of 3 mm and voxel size 3 mm^2^, 37 interleaved slices without gap, parallel to the AC-PC line, TR = 2000 ms, TE = 30 ms, fip angle of 75° and FOV of 210×210. On average, the scanning session lasted 45 min.

Data pre-processing was performed on BrainVoyager 21.4 software (Brain Innovation, Maastricht, The Netherlands). Pre-processing included slice-scan time correction, 3D head-motion correction and temporal high-pass filtering (2 cycles per run). Participants exceeding 6 mm of movement in any axis were excluded from further analysis (*n* = 5; 3 ASD and 2 TD). For the rest of the participants, we used motion as a confound predictor in the GLM model during the pre-processing phase. Data were normalized to a Montreal Neurological Institute (MNI) space and spatially smoothed using a Gaussian kernel with FWHM of 6 mm.

### Data Analysis

All statistical analysis was completed with the support of the Statistical Package for Social Sciences, version 26 (SPSS ®, Chicago, IL, USA) and the Brain Voyager 21.4 software (Brain Innovation, Maastricht, the Netherlands).

### Behavioural EcoSupermarketX data analysis

Several parameters were defined for the analysis of the EcoSupermarketX performance. Information about the EF that, in our perspective, is reflected by each EcoSupermarketX behavioural measure was extracted. The different behavioural measures/parameters defined were item errors, sequencing errors, time, distance and head rotation, which are detailed below:

- **Item errors**. The number of picked items of the EcoSupermarketX scenario that were not in the list of groceries divided by the number of items in the grocery list × 100 (e.g. select a toy, when the toy was not in the list).
- **Sequencing errors**. The number of picked items of the EcoSupermarketX scenario that were in the incorrect sequence in the list of groceries divided by the number of items in the grocery list × 100 (e.g. to select sausages before cereals, when the cereals were first in the list).
- **Total time**. Performance time (in seconds)—The time the participant was engaged in the execution of the task: looking for and grabbing the products that were in the grocery list (time elapsed from the end of the grocery list memorization to the last picked item).
- **Total distance**. Performance distance—The distance the participant goes through in the execution of the task: looking for and grabbing of the products that are in the grocery list).
- **Head rotation (orienting response)**. Sum of virtual head rotations by the participant (in degrees) during the time of execution of the task. This parameter aims to reflect attentional control, psychomotor and processing speed, planning and motor time.

Nonparametric statistics (Mann-Whitney *U* tests) were carried out for all statistical analyses to avoid biases due to deviations from normality and variance heterogeneity.

### Functional magnetic resonance data analysis

Statistical analysis was performed at the group level. For the GLM approach, the predictor’s model was obtained by convolution of the boxcar time course of each condition (no cue, non-social cue, social cue) with a two-gamma hemodynamic response function. The individual block duration was defined up to the participant’s response.

First, to compute a whole-brain statistical maps for group effect, an overlay random-effects (RFX) ANOVA with within-factors “Cue” (no cue, non-social cue, social cue) and between-factor “group” (ASD vs. TD) was conducted. These statistical maps were corrected for multiple comparisons using a cluster threshold method with a fixed *p*-value of .05 and an estimated minimum cluster extension of 70 contiguous voxels. The extension was estimated using Monte Carlo simulations (1000 iterations). This map revealed areas for which there are fundamental differences between groups irrespective of other factors.

In addition, for each identified region, post hoc *t*-tests were computed whenever significant group effects were found.

### Ethics statement

All the procedures were approved by the ethics committees from our University and Hospital Centre of Coimbra and were conducted in accordance with the Helsinki declaration. Written informed consent was obtained from the parents/guardians of all participants or, when appropriate, the participants themselves. Children and adolescents also gave oral informed consent.

### Community involvement

The community of local Patient Associations is involved in this study.

## Results

### Behavioural results

**Fig. 2:**
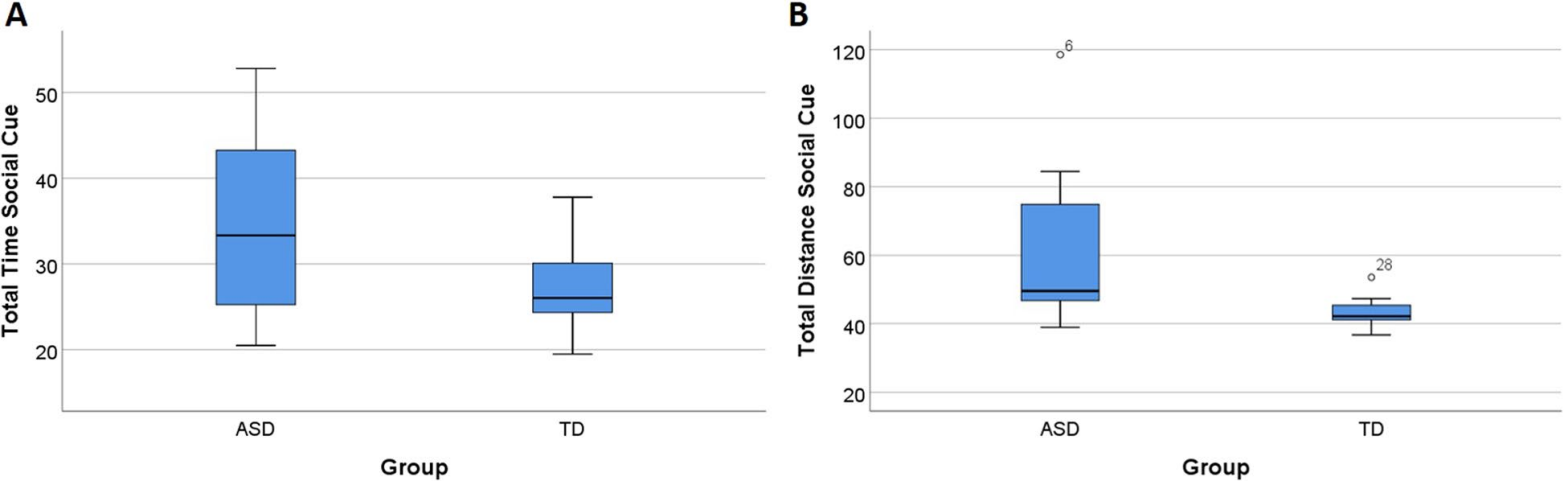
Performance across time and distance in social cue condition in ASD and TD groups. **A** Total time for ASD and TD groups for the social cue condition. **B** Total distance for ASD and TD groups for the social cue condition. Boxplots: central mark—median; edges of box—25th and 75th percentiles; whiskers—most extreme data points (minimum and maximum). Note. ASD, autism spectrum disorder, TD, typical neurodevelopment.

Behavioural analysis revealed that ASD and TD groups achieved similar performances in all EcoSupermarketX task parameters (*p* > .05, Mann-Whitney U), except for the items’ total time and distance in the social cue condition. The total time taken to perform the task was significantly higher for the ASD group (median **[**Mdn**]** = 34.5) than the TD group (Mdn = 26.9), *U* = 58.00, *p* = .041, *d* = .824. The same pattern was present in the total distance, with the ASD group (Mdn = 50.1) walking longer distances in the social cue condition than the TD group (Mdn = 43.0), *U* = 44.00, *p* = .007, *d* = 1.137. These results are summarized in Fig. 2.

Group performances were matched, since no significant statistical differences were seen, in other measured parameters: item errors, total sequencing errors and head rotation, both in social cue, non-social and no cue conditions.

### Eye-tracking measures

The time looking at the different AOIs of social or non-social relevance (head or arrow) that were related to the different types of cues (social and non-social, respectively) was compared between the two groups (ASD and TD).

A Mann-Whitney *U* test indicated no differences between the ASD and TD groups in the AOIs head (*U* = 99.00, *p* = 1.000) and Arrow (*U* = 114.00, *p* = .482).

### fMRI: whole-brain analysis of between-group effects in EcoSupermarketX task

**Fig. 3:**
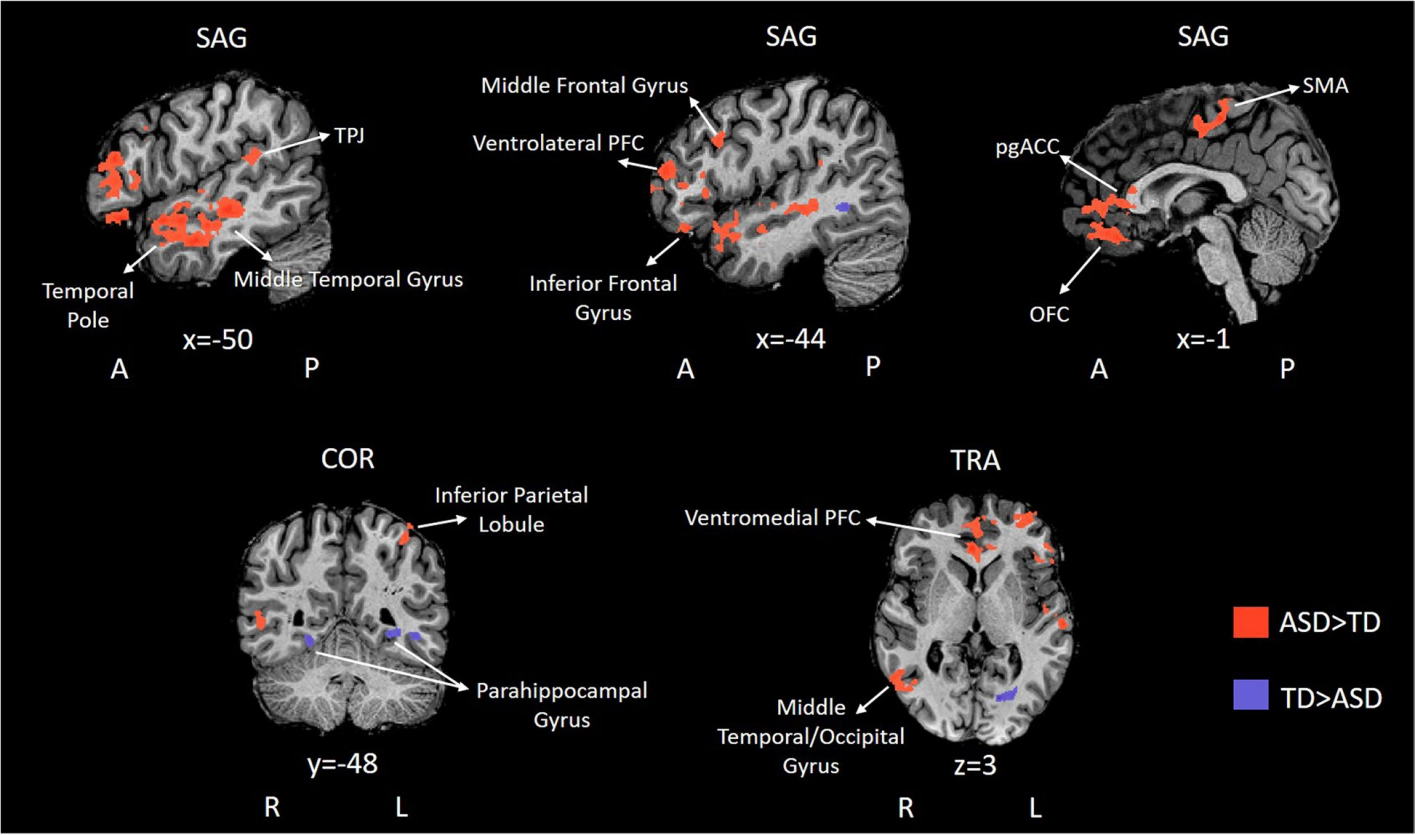
RFX ANOVA group effects for EcoSupermarketX task. Statistical maps from group analysis overlaid on sagittal, coronal, and transversal slices of a representative subject. Red clusters depict regions where BOLD activity was higher for individuals with ASD than TD. Blue clusters depict regions where BOLD activity was lower for individuals with ASD than TD. Slice locations are given in MNI coordinates. NOTE. ASD, autism spectrum disorder group, TD, typical neurodevelopment group, TPJ, temporal-parietal junction, PFC, prefrontal cortex, pgACC, pregenual anterior cingulate cortex; OFC, orbitofrontal cortex, SMA, supplementary motor area, A, anterior; P, posterior, R, right, L, left; SAG, sagittal plane; COR, coronal plane; TRA, transversal plane; MNI, Montreal Neurological Institute

The whole-brain RFX ANOVA revealed significant group effects in prefrontal (including ventromedial, orbitofrontal and anterior cortex), temporal (in particular the TPJ and the temporal pole) and visual areas. The differences between the pattern observed in ASD and TD groups are detailed in Table 2 (*F* ≥ 4.21; *p* < .05, corrected for multiple comparisons) and further highlighted in Fig. 3.

**Table 2:** Regions showing main effect of group in the whole brain RFX ANOVA analysis (*F* ≥4.21; *p*<.05, corrected for multiple comparisons): summary of RFX-GLM contrasts, outputs, and statistics

Region	BA	Peak MNI coordinates						No cue		Non-social cue		Social cue	
		*X*	*Y*	*Z*	No of voxels	*F*	*p*	*t*	*p*	*t*	*p*	*t*	*p*
ASD > TD													
RH middle temporal/occipital gyrus	37	53	− 55	− 2	1190	12.12	**.002**	4.006	**< .001**	4.969	**< .001**	3.943	**.001**
RH temporal pole	38	41	20	− 22	664	11.51	**.002**	3.242	**< .001**	4.812	**< .001**	3.668	**< .001**
RH primary somatosensory cortex/somatosensory association cortex/supramarginal gyrus	1/7/40	32	− 37	57	2726	16.02	**< .001**	5.114	**< .001**	6.073	**< .001**	5.820	**.001**
RH primary somatosensory cortex	1/4	19	− 31	57	710	10.13	**.004**	4.373	**< .001**	4.421	**< .001**	3.943	**.001**
Primary motor cortex													
L/RH supplementary motor area	6	− 2	− 28	54	2285	15.15	**< .001**	5.162	**< .001**	5.721	**< .001**	5.467	**< .001**
L/RH ventromedial prefrontal cortex/pregenual anterior cingulate cortex	32	− 6	50	− 8	4439	19.04	**< .001**	4.575	**< .001**	5.611	**< .001**	3.690	**< .001**
Orbitofrontal cortex	10/11												
LH middle frontal gyrus	10	− 33	51	− 1	1979	13.69	**.001**	3.509	**.002**	3.391	**.003**	3.692	**.001**
LH middle temporal gyrus	21/22	− 60	− 19	5	10826	22.10	**< .001**	5.405	**< .001**	7.688	**< .001**	4.703	**< .001**
LH middle frontal gyrus	8/9	− 30	24	42	1347	14.18	**< .001**	4.040	**< .001**	4.389	**< .001**	4.040	**.001**
LH inferior parietal lobule	40	− 38	− 49	53	805	12.02	**.002**	5.114	**< .001**	6.073	**< .001**	5.820	**< .001**
Claustrum		− 37	− 2	− 18	618	10.42	**.003**	2.152	**< .001**	4.530	**< .001**	3.367	**< .001**
LH inferior frontal gyrus/ventrolateral prefrontal cortex	45/46/47	− 50	− 2	− 18	3552	15.38	**< .001**	4.512	**< .001**	5.440	**< .001**	3.851	**.001**
LH temporal parietal junction	22/40	− 60	− 27	21	1937	19.70	**< .001**	5.108	**< .001**	4.747	**< .001**	4.928	**< .001**
TD > ASD													
RH parahippocampal gyrus	19	26	− 49	− 7	586	11.18	**.002**	− 4.008	**.001**	− 4.318	**.001**	− 4.198	**.001**
LH parahippocampal gyrus	18/19	− 16	− 72	1	1810	15.41	**< .001**	− 8.547	**< .001**	− 9.091	**< .001**	− 8.479	**< .001**

*Note.* Brain regions showing significant whole-brain RFX-ANOVA group effect (*p* < .05, corrected for multiple comparisons) and ROI-based GLM-RFX contrasts for group differences between responses to Social, Non-Social and No Cue conditions. Positive *t-*tests indicate higher *β* values for the ASD group than for TD. Negative *t*-tests indicate higher *β* values for the TD group than for ASD. *X*, *Y* and *Z* represent MNI coordinates. Significant comparisons are marked in bold. *BA*, Brodmann area; *ASD*, autism spectrum disorder group; *TD*, typical neurodevelopment group, *RH*, right hemisphere, *LH*, left hemisphere

Region of interest-based post hoc *t*-tests confirmed that across social cognition, executive and saliency networks ASD showed higher BOLD activity when compared with TD in response to EcoSupermarketX task conditions. This included regions such as the middle frontal gyrus, ventrolateral PFC, ventromedial PFC, orbitofrontal cortex (OFC), inferior frontal gyrus, middle temporal cortex, temporal pole, TPJ, ACC and supramarginal gyrus. The same analysis revealed higher BOLD activity for TD compared to ASD only in the parahippocampal gyrus (for further details on statistical significance, see Table 2).

## Discussion

We investigated the neural correlates of executive functioning when ASD and healthy participants engage in a demanding, goal-oriented ecological task, where social and non-social cues are present. We used markers of EF under distinct task constraints, with explicit manipulations of the types of cues that improve task performance. We also monitored visual attention to specific AOI’s social (avatar’s head) and non-social (arrow) cues, while shopping.

### Preserved ASD performance for non-social conditions

We found that, in the absence of a cue or with the non-social cue, groups did not differ. ASD group only performed worse in two specific behavioural measures of the social cue condition: total time and distance taken to do the task. In fact, participants with ASD did not differ from TD in item errors, sequencing errors and total head rotation in all conditions, corroborating the notion that groups were overall matched in terms of performance.

### Higher ASD recruitment of executive, saliency and social networks during EcoSupermarketX

When performing this virtual ecological daily living task—going shopping, we found differential activation across three main networks: social, executive, and saliency circuits. Accordingly, we found higher activity by the ASD group when compared to TD in areas that are pivotal in social brain circuits, namely the TPJ, ventromedial PFC and inferior parietal lobule. Importantly, the TPJ, a part of the ventral attention system, has also been implicated in social and emotional processing [56–59], including joint attention [60–62], attentional reorienting to salient cues [63] and visually triggered shifts of attention that share common neural substrates with gaze perception [64]. Our task had three blocks of increasing executive load and incorporated social and non-social cues, with different degrees of saliency, which is ideally suited to recruit attentional, executive and saliency networks. These aspects are quite important in our task design and are related to core characteristics of individuals with ASD, as corroborated by our previous behavioural study [55]. Moreover, it is also known that the TPJ is most reliably activated when participants engage in tasks involving the inference of goals or end states for described actions [65].

Larger recruitment of areas of the executive network was also found in individuals with ASD during EcoSupermarketX, namely in the middle frontal gyrus, and also pregenual ACC which is part of the salience network.

The only region that showed reduced activity in individuals with ASD as compared to controls was the parahippocampal gyrus. It is known to be involved in scene recognition, spatial navigation and memory encoding and retrieval [66], which are required to perform the presented task and it is known to be a strength in the cognitive profile of individuals with ASD.

We interpret our results in light of the notion that the over-recruitment observed for the three main networks reflects the large cognitive burden that this type of ecological task poses to participants with ASD. Given that individuals with ASD are quite functional in terms of scene recognition and navigation, this might explain why parahippocampal under-recruitment occurs in comparison to controls. In other words, relative proficiency leads to less brain activation because fewer resources are needed. A study comparing TD adolescent girls before and after practice on a visual-spatial problem-solving computer game, Tetris, showed that brain activity decreases after practice [67]. Additionally, a recent study [68] showed that no impairments emerged in visuospatial working memory in individuals with ASD, as compared with TD individuals, which corroborates our previous work [69], showing that this ability is relatively spared. This is in line with the notion of peaks and valleys of performance in individuals with ASD.

Importantly, this pattern of simultaneous recruitment of three major networks (and lower activity for the parahippocampal gyrus) was found for the three conditions in the task. As the final performance of the task is quite similar between the groups, we may speculate that the ASD group maintains a higher neural effort, as a putative compensatory mechanism, to reach similar performance levels as the TD group. Although neural compensation mechanisms are still controversial, this neurobehavioural experiment showed that ASD correctly used the structured cues as TD does in goal-oriented tasks.

Considering that shopping is a challenging task for the majority of individuals with ASD and our results showed they are engaged in an extra mental effort in the executive, saliency and social brain networks to achieve accurate performance; we may assume that they had to make a more focused effort, at the attentional, executive and emotional levels than the TD group. This would explain why there is higher activity in the OFC, an area that is part of the limbic system, being involved in the control emotional reactions in certain social situations, as well as the process of decision-making and self-control [70]. OFC has also been thought to contain a representation of task space, which is especially critical for behaviour when states are unobservable from sensory input [71].

Similar performance scores and different patterns of brain activation relative to controls had been reported in functional neuroimaging studies but with distinct significance: these studies focused on response inhibition in adults with ASD [28, 72, 73]. Kana et al. [72] found that the adults with ASD relative to controls had decreased activity, namely the in ACC, while Schmitz et al. [28] found increased activity for the ASD group in several brain regions known to be involved in response inhibition, including the inferior frontal gyrus and the OFC during a simple Go/NoGo task using non-social stimuli. However, most of these studies differ from ours in two very important aspects: ecologic setup and multiple task demands (social, attentional, executive) that are inherent to our paradigm.

Our non-intellectually disabled individuals with ASD understood and performed the shopping task as the TD group and presented little performance differences and no changes in the eye-tracking measures when compared to the TD group. Our participants with ASD used the structured cues as TD in a goal-oriented manner.

Concerning the limitations of our study, they are reflected in its small sample size.

## Conclusions

In conclusion, our functional imaging results are consistent with the notion that hyperactivation of particular networks may provide a compensatory mechanism to preserve performance [73] in contrast with scene recognition parahippocampal networks. It is remarkable that for the first time in the current study this was observed simultaneously across executive, saliency and social cognition networks, which reinforces the dysfunction of this pivotal pathway in individuals with ASD, a network signature that may represent a possible intervention target.

## Data Availability

The datasets used and/or analysed during the current study are available on reasonable request.

## Abbreviations

3D: Three-dimensional
A: Anterior
ACC: Anterior cingulate cortex
ADI-R: Autism Diagnostic Interview—Revised
ADI-R L/C: ADI-R Language/Communication
ADI-R RB/I: ADI-R Repetitive Behaviours/Interests
ADI-R RSI: ADI-R Reciprocal Social Interactions
ADOS: Autism Diagnostic Observation Schedule
ADOS COM: ADOS Communication
ADOS SI: ADOS Social Interaction
AOI: Area of interest
ASD: Autism spectrum disorder
BA: Brodmann area
BOLD: Blood-oxygen-level-dependent
CA: Chronological age
CEN: Central executive network
COR: Coronal plane
DLPFC: Dorsolateral prefrontal cortex
DSM-5: Diagnostic and Statistical Manual of Mental Disorders 5
EF: Executive functioning
EPI: Echo-planar imaging
F: Female
fMRI: Functional magnetic resonance imaging
FOV: Field of view
FSIQ: Full-scale intelligence quotient
GLM: General linear model
IPL: Inferior parietal lobule
IQ: Intelligence quotient
IQR: Interquartile range
L: Left
LCD: Liquid crystal display
LH: Left hemisphere
M: Male
Mdn: Median
MFG: Middle frontal gyrus
MNI: Montreal Neurological Institute
MPFC: Medial prefrontal cortex
MPRAGE: Magnetization-prepared rapid acquisition gradient echo
MRI: Magnetic resonance imaging
OFC: Orbitofrontal cortex
P: Posterior
pgACC: Pregenual anterior cingulate cortex
PCC: Posterior cingulate cortex
PFC: Prefrontal cortex
PHG: Parahippocampal gyrus
PIQ: Performance intelligence quotient
R: Right
RH: Right hemisphere
RFX: Random-effects
SAG: Sagittal plane
SMA: Supplementary motor area
SPL: Superior parietal lobule
SPSS: Statistical Package for Social Sciences
STS: Superior temporal sulcus
TD: Typical neurodevelopment
TE: Echo Time
TPJ: Temporo-parietal junction
TR: Repetition time
TRA: Transversal plane
VIQ: Verbal intelligence quotient
vlPFC: Ventrolateral prefrontal cortex
vmPFC: Ventromedial prefrontal cortex
WAIS-III: Wechsler adult intelligence scale—Third Edition
WISC-III: Wechsler intelligence scale for children—Third Edition
